# A new less invasive surgical technique in the management of acute Achilles tendon rupture through limited-open procedure combined with a single-anchor and “circuit” suture technique

**DOI:** 10.1186/s13018-018-0895-x

**Published:** 2018-08-10

**Authors:** Hao Zhang, Pei-Zhao Liu, Xin Zhang, Chen Ding, Hao-Chen Cui, Wen-Bin Ding, Ren-Kai Wang, Da-Jiang Wu, Qiang Wei, Sheng Qin, Xue-Lin Wu, Da-Ke Tong, Guang-Chao Wang, Hao Tang, Fang Ji

**Affiliations:** Department of Orthopaedics, Changhai Hospital, Second Military Medical University, No 168 Changhai road, Shanghai, 200433 China

**Keywords:** Achilles tendon, rupture, acute, Suture anchors, “Circuit” suture technique, Minimally invasive surgical procedures

## Abstract

**Background:**

Traditional incision repair and minimally invasive repair for acute Achilles tendon repair have limitations. This study aimed to present our series of 23 patients with acute Achilles tendon rupture that was repaired using two small incisions to assist the anchor repair of the tear and a new “circuit” suture technique.

**Methods:**

This was a retrospective study of 23 patients with acute Achilles tendon rupture treated with the new technique at Changhai Hospital between January 2015 and December 2016 and followed up for 14–33 months. Clinical outcome was assessed using the AOFAS, Leppilahti, and Arner-Lindholm scores. Complications, range of motion (ROM), and time to return to work and light sport activity were assessed.

**Results:**

The AOFAS score was 85–96 at 3 months and 92–100 at 12 months. The 3-month ROM was 27°–37°, and the 12-month ROM was 36°–48°. The Leppilahti score was 85–95 at 3 months and 90–100 at 12 months. The recovery time of the patients was 10–18 weeks. The postoperative recovery time to exercise was 16–24 weeks. There was only one case of deep venous thrombosis. According to the Arner-Lindholm assessment criteria, patient outcomes were rated as excellent in 20 (87.0%) cases, good in three (13.0%) cases, and poor in 0 cases. The excellent-to-good rate was 100%.

**Conclusion:**

The limited-open procedure combined with a single-anchor and “circuit” suture technique could be used to repair torn Achilles sites, with a low occurrence of complications. This new and minimally invasive technique could be an alternative in the management of acute Achilles tendon rupture.

## Background

Acute Achilles tendon rupture is a common sports-related injury [1]. This rupture mainly occurs in males aged 30 to 50 years [2–4], with a male-to-female ratio of 3:1 [1]. Acute Achilles tendon rupture may be caused by various etiological factors, the most common being sudden violence during exercise [5]. In addition, patients with degeneration of the Achilles tendon [6], long-term steroid use [7, 8], or use of quinolone antibiotics [9] are at higher risk of rupture.

The purpose of treatment for acute Achilles tendon rupture is to restore the continuity of the Achilles tendon and normal function of the triceps surae, but the best approach remains controversial [10–13]. Conservative treatment is often used for patients who have a sedentary lifestyle and severe medical conditions, and in those who are reluctant to undergo surgery; these patients have a slow recovery and high risk of re-rupture [14]. Surgical repair can reduce the fixation time, allow for early load and functional exercise, and decrease the risk of re-rupture [15]. Therefore, surgical repair is still the main treatment for acute Achilles tendon rupture, especially in young patients and athletes [6], but there is some concern over the risk of complications and there is no consensus on the best surgical approach [16].

The traditional method of incision repair can provide strong repair, with a low re-rupture rate of 1.4–2.8% [17], but a high incidence of soft tissue complications (11–34.1%) [18]. Minimally invasive and transdermal repair methods result in less trauma and can significantly reduce the risk of soft tissue complications [19], but due to generally unexposed torn sites, there is a concern that some percutaneous methods may result in poor involution and weaker biomechanical resistance of the torn sites compared with traditional incision repair, resulting in a higher risk of re-rupture [20, 21]. Moreover, the sural nerve injury rate is relatively high (0–10%) [22].

In view of the issues with traditional incision repair and minimally invasive repair, we designed an approach (based on the technique by Amlang et al. [23]) that uses two small incisions to assist the anchor repair of the torn sites based on tendon-bone suture and a new “circuit” suture technique. The anchor was used to enhance the mechanical strength of the torn site after repair, and the traditional large incision was reduced to two small incisions. This method limits the damage to the skin and could be expected to reduce the occurrence of complications while ensuring the repair strength of the tear.

Therefore, the objective of the present study was to present our series of 23 patients with acute Achilles tendon rupture that was repaired using this novel approach. This method could improve the outcomes of Achilles tendon repair.

## Methods

### Subjects

This was a retrospective study of consecutive patients treated for Achilles tendon rupture from January 2015 to December 2016, at Changhai Hospital. Twenty-three patients (23 ruptured tendons) were eligible. All patients presented with sudden foot heel pain, difficulty walking, and being unable to lift heel. They were all admitted to the hospital.

The indications for the new method of Achilles tendon repair were (1) patients with acute Achilles tendon rupture, (2) within 2 weeks of injury, (3) palpable gap at the torn sites of the Achilles tendon and positive Thompson test, (4) B-mode ultrasound showing that the Achilles tendon was completely ruptured, and (5) the distance between the torn sites and the calcaneal insertion was within 2–6 cm.

The exclusion criteria were (1) open Achilles tendon rupture (*n*\4); (2) incomplete clinical data (*n* = 3); or (3) history of rheumatoid diseases, long-term use of steroids or quinolone, older patients (> 65 years of age) with comorbidities that can affect healing (such as diabetes), or those unwilling to undergo surgery (*n* = 5).

This study was approved by the hospital’s ethics committee. All patients signed a surgical consent form.

### Surgery

Surgery was performed under either spinal anesthesia or sciatic nerve block anesthesia. The patient was placed in the prone position. The calf was elevated to provide knee flexion of 20°–30°, between a neutral position and maximal plantar flexion. Tourniquets were not used. General disinfection pads were used in the operative field. A preoperative dose of 2 g of cefotiam was intravenously infused. All surgeries were performed by the same surgeon.

**Fig. 1:**
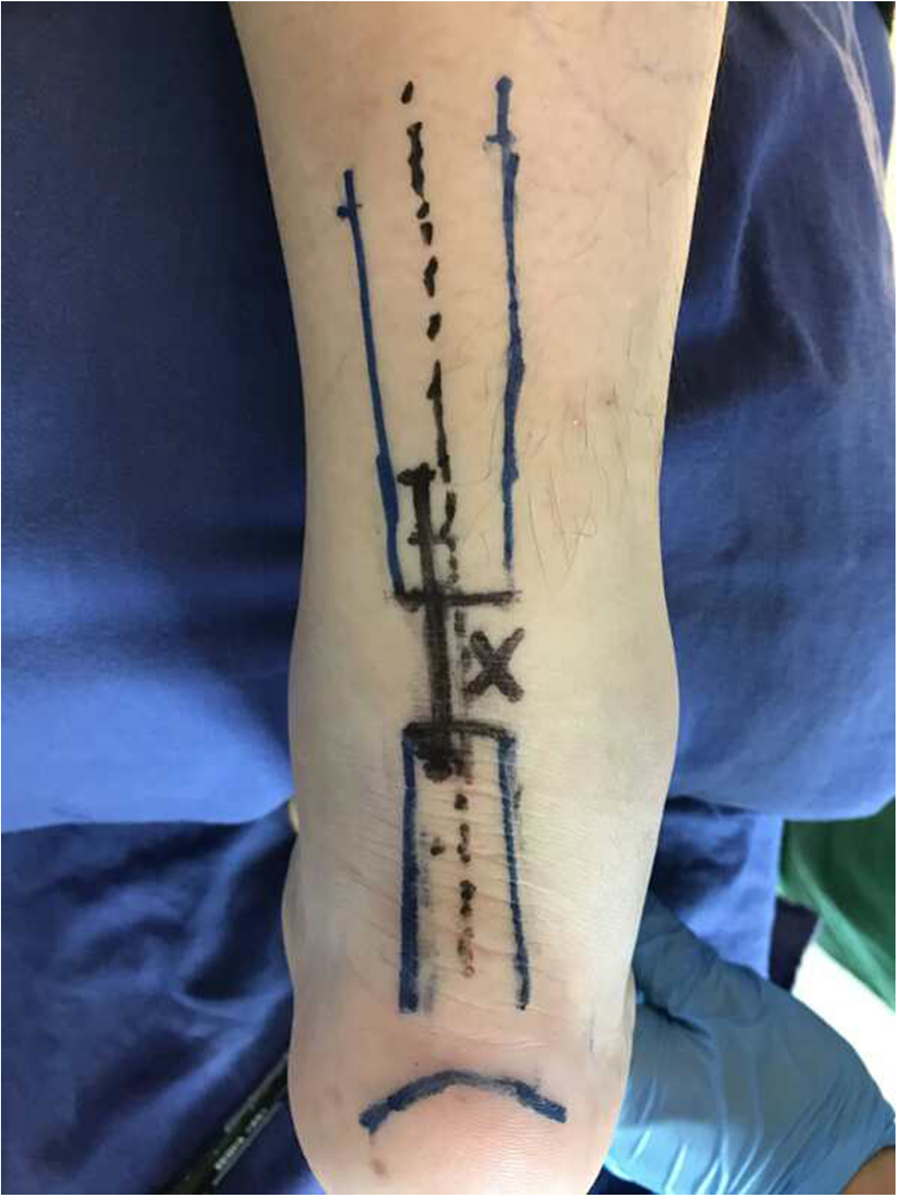
Surgical planning. The blue arc in the distal part refers to the superior margin of the calcaneum. The blue rectangles refer to the profile of the Achilles tendon. The dotted black line refers to the central line of the Achilles tendon. The x is where the tear is. The solid black line refers to the proximal medial incision

**Fig. 2:**
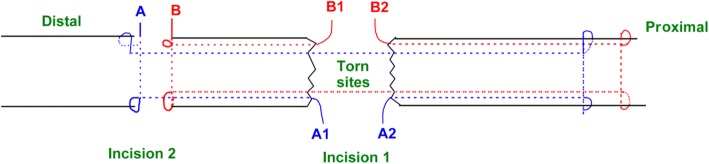
“Circuit” suture. Two groups of sutures were used to repair the torn sites. (A) Entry point for the first suture. (A1) Exit point for the first suture. (A2) Exit point for the second suture. (B) Entry point for the third suture. (B1) Exit point for the third suture. (B2) Exit point for the fourth suture

**Fig. 3:**
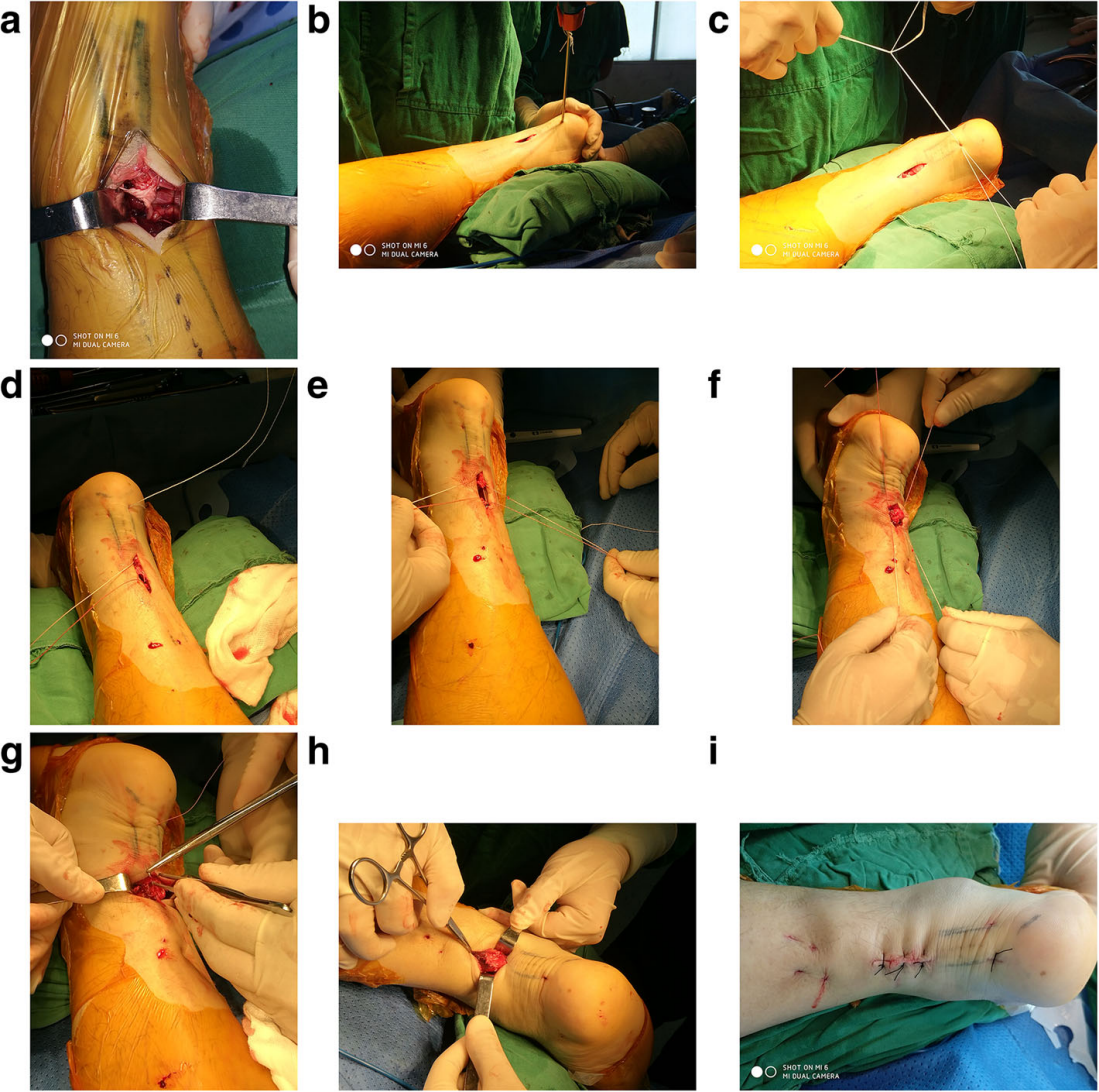
Operation procedure. **a** The torn end was revealed, which was a caudal tear in this case. **b** A rivet was screwed into the calcaneum. **c** The two sets of rivet suture were knotted. **d** The first backstitch. **e** The second backstitch in a different layer. **f** The two sets of suture were knotted in the plantar flexion position. **g** Verification of the tension at the torn end to appropriately reinforce the suture. **h** Diagram of the broken end after stitching. **i** Postoperative incision

Figure 1 presents the preoperative planning. Figure 2 presents the “circuit” suture schematic. Figure 3 presents the operative process. The torn ends of the tendon were located, and a longitudinal incision of about 3 cm (the proximal end was slightly longer than the distal end) was made at the anteroposterior tendon along the torn sites, by in turn cutting the skin, subcutaneous tissue, and Achilles tendon sheath membrane in order to expose the torn ends. The ruptured ends were cleared of blood clots, trimmed, and then held with a Kocker clamp for the suture. A longitudinal incision of about 0.5 cm was made at about 0.5 cm above the calcaneal insertion. A small amount of stripping was done at the calcaneal insertion in the posterosuperior tubercle of the calcaneus, and a suture anchor (Twinfix 5 mm, Smith & Nephew, London, UK) was inserted perpendicularly into the calcaneus. The handle was removed, and the two groups of sutures were knotted separately. The two groups of sutures (#2 Ultrabraid non-absorbable sutures, Smith & Nephew, London, UK) at the anchor tail were used to repair the torn sites at different layers by using a “circuit” technique, in which the shape of a square within a square is formed (which is similar in shape to the symbol that represents the Chinese word “circuit”). From the starting point, a large round needle with a strand suture that could go through plastic was transversely inserted through the distal tendon tissue to the other side. After being inserted along the point, the needle was vertically inserted through the first suture along the distal tendon tissue to the distal torn ends, forming half of the square within a square shape. At the side of the anchoring nail, another large round needle was passed vertically through the distal tendon tissue and the torn sites to the proximal tendon tissue, followed by piercing through the skin in an appropriate location.

Then, once again, the needle was inserted through the second suture that was placed transversely through the tendon tissue to the other side and then pierced the skin. The needle was longitudinally inserted through the second suture along the proximal tendon tissue to the proximal torn ends, forming the other half of the square within a square. These two sutures were knotted to form the whole square within a square shape. Similarly, another set of sutures on the anchor were attached to the large round needle to repair the torn sites of the Achilles tendon at different layers. The needle was inserted through the third suture along the distal tendon tissue to the distal torn ends, forming half of the square within a square. Then, the needle was inserted through the fourth suture transversely through the distal tendon tissue to the other side, followed by insertion through the fourth suture along the torn ends, and then inserted transversely through the proximal tendon tissue to the other side. Finally, the needle was vertically inserted through the fourth suture along the proximal tendon tissue to the other side of the proximal torn ends. These two sutures were knotted when the foot was placed in plantar flexion position in order to make the torn ends contact and maintain the tendon at an appropriate tension. Tension and continuity at the torn site was examined, and reinforcement suturing was performed as necessary. Therefore, two circuit sutures were placed at different layers in the tendon tissue using 2–0 absorbable sutures (Ethibond, Ethicon Endo-Surgery, Cincinnati, OH, USA). The knots of the anchor sutures were wrapped in the Achilles tendon. The Achilles tendon sheath membrane was intermittently stitched before closing the incisions one by one. Sterile dressings were applied.

### Postoperative management

The patients were hospitalized for 1–3 days according to incision healing status, and they received anti-inflammatory drugs. Enoxaparin (40 mg, day 1, subcutaneous injection) was administrated to prevent deep vein thrombosis. The dressings were changed as required, and the patient was discharged with the ankle fixed by a plaster and anti-inflammatory drug prescription. Fixation was achieved using tube-fixing plaster below the knee in plantar flexion of 20°–30° or using functional braces. The patients were allowed to walk with crutches, without any weight bearing on the affected leg. The first plaster and stitches were removed 2 weeks postoperatively, and a second plaster was applied with the foot in neutral position. The plaster was removed after 4 weeks, and the patients were encouraged to carry out ankle dorsiflexion and plantar flexion function exercises. After 6 weeks, the patients were allowed to wear normal shoes with 2-cm high heels and gradually carried out heel raising exercises and walked without crutches. After 8 weeks, stretching exercises with a progressive increase in weight bearing were permitted by 5-N increments. The patients were helped to correct their limping gait and gradually return to their normal daily activities.

### Outcome measurements

Time from injury to surgery, operation time, proximal and distal incision size, and intraoperative blood loss were recorded. During follow-up, the occurrence of soft tissue complications (whether the incision healed by primary intention, incision infection, skin necrosis, deep infection, scarring, etc.), sural nerve injury, re-rupture, and deep venous thrombosis were recorded. The patients’ postoperative time of return to work and light sports (normal gait while walking and running, good muscle strength, and could swim) was also recorded. The American Orthopedic Ankle Association (AOFAS) score [24], the Leppilahti score [25], the circumference differences of the two calves, and the range of motion (ROM) of the repaired ankle joint were recorded at 3 and 12 months. The circumferences of the two calves were measured using a measuring tape centered at the midpoint of the gastrocnemius muscle. The dorsiflexion and plantar flexion range of the operated ankle were assessed qualitatively and quantitatively using the Arner-Lindholm assessment criteria.

### Statistical analysis

Descriptive statistics were used to describe the general patient data. SPSS 19.0 (IBM, Armonk, NY, USA) was used for analysis. Normally distributed data were expressed as mean ± standard deviation; otherwise, data were expressed as median and range.

## Results

### Baseline characteristics of the patients

The baseline characteristics of the 23 patients are shown in Table 1. In this study, 19 patients were male and four were female. They were aged 23–57 years (mean, 39.0 ± 9.3 years). Among them, there were 14 left foot injuries and nine right foot injuries. The causes of injury were sports in all cases (basketball, *n* = 10; football, *n* = 7; badminton, *n* = 3; and running, *n* = 3). B-mode ultrasound showed complete rupture in all cases at 3.2–4.4 cm from the calcaneal insertion. The time between injury and operation was 1–3 days, with a median time of 2 days (Table 2).

**Table 1:** Characteristics of the patients

Variable	Study subjects (*n* = 23)
Age, years (mean ± SD)	38 ± 8
Male (*n*, %)	19 (82.6%)
Weight, kg (mean ± SD)	72 ± 10
Height, cm (mean ± SD)	173 ± 6
Body mass index, kg/m^2^ (mean ± SD)	24.0 ± 2.2
Injured side (*n*, %)	
Left	14 (60.9%)
Right	9 (39.1%)
Cause of injury (*n*, %)	
Basketball	10 (43.5%)
Football	7 (30.5%)
Badminton	3 (13.0%)
Running	3 (13.0%)
Tear site (distance from the calcaneal insertion), cm (mean ± SD)	3.8 ± 0.3

*SD* standard deviation

**Table 2:** Perioperative indexes

ID	Time from injury to surgery (days)	Operation time (min)	Proximal incision (cm)	Distal incision (cm)	Intraoperative blood loss (ml)
1	2	30	3	1	30
2	1	26	2.5	0.5	30
3	2	22	3	0.8	25
4	2	28	3.2	0.5	20
5	2	24	2.8	0.6	35
6	3	28	2.8	0.8	30
7	2	26	3.2	0.5	30
8	1	20	2.7	0.5	25
9	3	30	3.2	0.8	30
10	2	25	3	0.6	25
11	1	22	3.2	0.5	20
12	2	24	3.5	0.8	35
13	1	28	3.2	0.8	30
14	1	32	2.8	0.5	35
15	2	26	3.2	0.5	30
16	2	28	2.8	0.6	25
17	1	34	3.2	0.8	40
18	1	26	2.8	0.5	20
19	2	28	3	0.6	30
20	2	32	3.5	0.8	40
21	1	30	3.3	0.6	45
22	2	36	2.8	1	50
23	3	30	3	0.5	30
Mean ± SD	1.8 ± 0.7	27.6 ± 3.9	3.0 ± 0.3	0.7 ± 0.2	31 ± 8
Median	2	28	3	0.6	20
Range	1–3	20–36	2.5–3.5	0.5–1.0	20–50

*SD* standard deviation

### Surgical outcomes

The perioperative indexes are shown in Table 2. The time between injury and operation was 1–3 days, with a median time of 2 days. The operation time was 20–36 min, median of 28 min. The length of the proximal incision was 2.5–3.5 cm, mean of 3.0 ± 0.3 cm. The length of the distal incision was 0.5–1 cm with an average of 0.7 ± 0.2 cm. Intraoperative blood loss was 20–50 ml, with an average of 31 ± 8 ml.

### Clinical outcomes

**Fig. 4:**
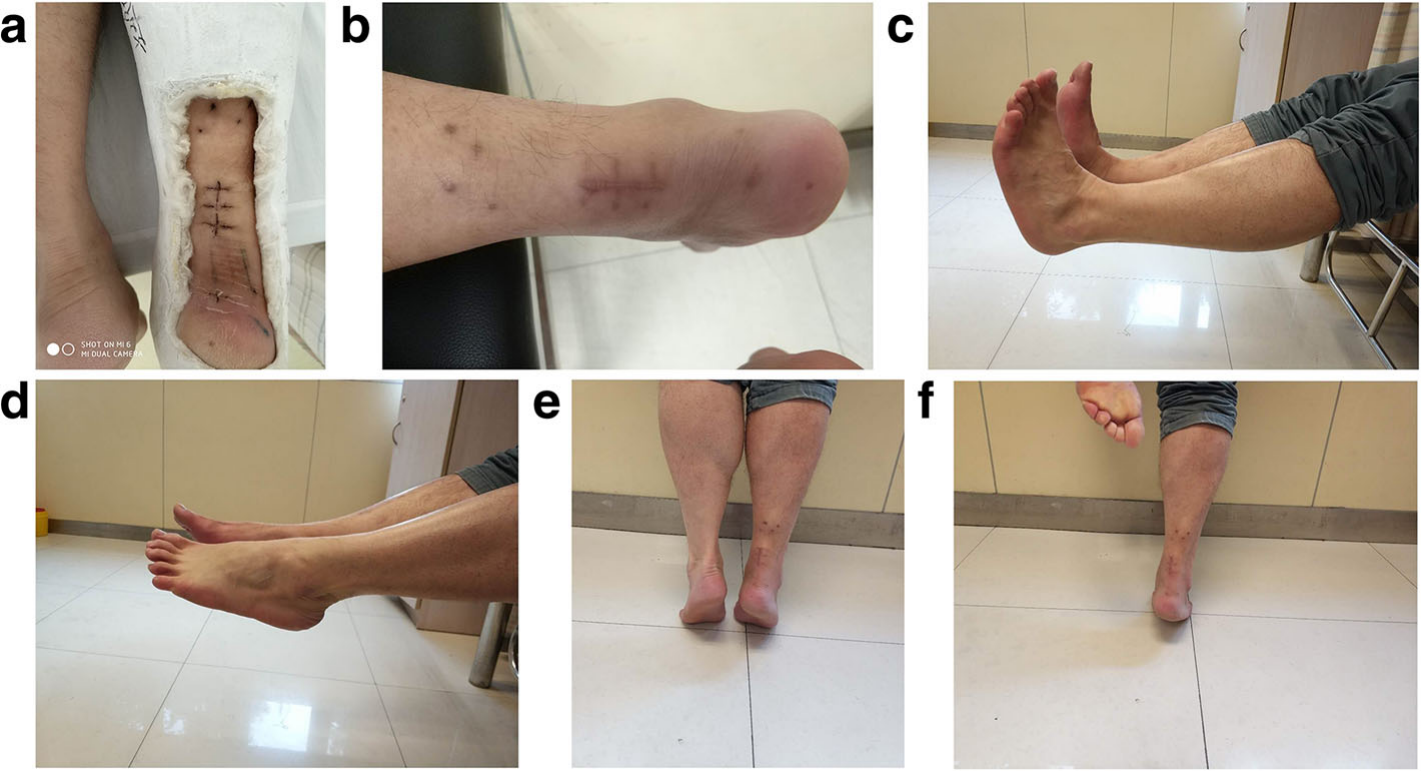
Postoperative recovery. **a** Stitches were removed 2 weeks postoperatively, followed by plaster fixation. **b** Incision at 3 months postoperatively. **c** Dorsal extension at 3 months postoperatively. **d** Plantar flexion at 3 months postoperatively. **e** Heel raising at 3 months postoperatively. **f** Heel raising of the affected foot at 3 months postoperatively

The 23 patients were followed up for 14–33 months (median, 22 months). The follow-up results are shown in Table 3 and Fig. 4. There were no cases of soft tissue complications. All incisions healed by first intention, without incision infection or split, skin necrosis, and deep infection. There was no obvious adhesion between the repair site and the skin, except for one patient during follow-up, with a large scar and poor mobility. This might be due to the incomplete enclosure of the torn ends by the tendon sheath. During follow-up, complications such as Achilles tendon re-rupture and sural nerve injury did not occur. There was only one case of deep vein thrombosis. The AOFAS score of the affected side was 85–96 (median, 92) at 3 months and 92–100 (median, 96) at 12 months. The 3-month ROM was 27–37 (median, 32), and the 12-month ROM was 36–48 (median, 42). The Leppilahti score was 85–95 (median, 90) at 3 months and 90–100 (median, 95) at 12 months. The recovery time of the patients was 10–18 weeks (median, 14 weeks). The postoperative recovery time to light activity was 16–24 weeks (median, 18 weeks). At 12 months, any difference in calf circumference between the operated and healthy sides was < 1.0 cm, indicating no significant muscle atrophy due to limb inactivity.

**Table 3:** Postoperative follow-up index

ID	Follow-up (months)	Complications	3-month AOFAS	12-month AOFAS	3-month ROM	12-month ROM	3-month LS	12-month LS	3-month calf circumference difference (cm)	12-month calf circumference difference (cm)	Time to work (weeks)	Time to light sports (weeks)
1	18	No	95	98	37	45	95	100	0.8	0.5	10	16
2	29	No	93	98	36	44	90	100	0.8	0.6	12	16
3	18	No	90	94	33	40	90	95	1.2	0.8	14	18
4	20	No	92	96	34	43	90	95	1	0.6	14	20
5	28	No	91	98	32	44	95	100	0.8	0.5	13	16
6	24	No	90	94	30	40	90	95	1.2	0.8	14	20
7	22	No	91	95	32	42	90	95	1.2	0.8	13	18
8	28	No	96	100	34	42	95	100	0.6	0.2	11	16
9	26	No	92	95	32	43	90	95	1.2	0.8	12	18
10	24	No	85	92	27	36	85	90	1.8	1.2	18	24
11	26	No	95	100	37	48	95	100	0.5	0.2	12	16
12	33	No	94	98	34	45	95	100	1	0.6	12	16
13	24	No	90	94	28	38	90	95	1	0.8	12	18
14	22	DVT	92	95	30	40	90	95	1	0.8	14	20
15	14	No	92	96	32	42	90	95	1	0.8	14	19
16	21	No	93	97	32	42	90	95	0.8	0.6	14	20
17	18	No	92	96	30	38	95	95	1	0.6	15	20
18	16	No	93	97	33	44	95	95	1	0.6	14	18
19	23	No	93	96	30	42	95	95	1.2	0.8	13	18
20	17	No	91	94	30	40	95	95	1.2	0.8	15	21
21	16	No	90	93	28	40	90	90	1.4	1	16	22
22	18	No	93	96	32	44	95	95	1.2	0.6	14	18
23	20	No	93	97	32	43	95	95	1.2	0.6	14	19
Mean ± SD	22 ± 5	–	92 ± 2	96 ± 2	32 ± 3	42 ± 3	92 ± 3	96 ± 3	1.0 ± 0.3	0.7 ± 0.2	13 ± 2	19 ± 2
Median	22	–	92	96	32	42	90	95	1.0	0.6	14	18
Range	14–33	–	85–96	92–100	27–37	36–48	85–95	90–100	0.5–1.8	0.2–1.2	10–18	16–24

*AOFAS* American Orthopedic Foot and Ankle Society, *ROM* range of motion, *LS* Leppilahti score

The recovery of ankle dorsiflexion, plantar flexion, and heel raising function were good. Operated ankle joint dorsiflexion and plantar flexion were 20–30°. According to the Arner-Lindholm assessment criteria, patient outcomes were rated as excellent in 20 (87.0%) cases, good in three (13.0%) cases, and poor in 0 cases. The excellent-to-good rate was 100%.

## Discussion

In the present study, we combined the advantages and disadvantages of traditional incision repair and percutaneous repair and designed a minimally invasive repair method (based on the technique by Amlang et al. [23]) that uses two small incisions combined with single-anchor and “circuit” suture technique. This new technique aims to firmly repair the tear and reduce the incidence of Achilles tendon re-rupture, soft tissue complications, and sural nerve injury. The results of this series of 23 patients suggest that it could be possible to achieve good repair outcomes with no complications after a median of 22 months of follow-up. All 23 patients were rated as excellent/good according to Arner-Lindholm assessment criteria, similar to the outcomes of a previous study that presented a similar minimally invasive surgical repair for acute ruptures of the Achilles tendon [26]. Indeed, Maffulli et al. [26] showed that 80% of their patients returned to their preoperative level of activity, while 10% only showed lower activity.

The method used in this study is based on the traditional incision repair, but with improvements. The traditional direct cut was changed into a limited incision to avoid incision of those areas of the skin most prone to wrinkle, which significantly reduces the degree of post-suture tension and the occurrence of skin folds and reduces the incidence of soft tissue complications. Meanwhile, in order to reduce the occurrence of Achilles tendon re-rupture and sural nerve injury, the torn ends of the tendon were exposed intraoperatively, and an anchor was screwed into the appropriate position of the calcaneus in the distal small incision. The circuit technique was used for suture repair by tail suture of an anchor at different layers of the tendon tissue, which is different from the method by Guillo et al. [27], who pull the torn ends together and place the sutures in the paratendon tissue, while we place the sutures directly in the tendon. The circuit technique is based on the principle of tendon-bone suture, improved Kessler suture [28], and other previous techniques [23]. The needle is inserted from the distal small incision, and two circuit Chinese characters are sutured in the different layers of tendon tissue. In addition, the force of the anchor is used to enhance the biomechanical strength after the repair of the torn ends, reducing the chance of Achilles tendon re-rupture. This method shares some similarities with previously published methods [27, 29, 30], but the suture technique used here was different.

With the method used in this study, the incidence of soft tissue complications was significantly reduced, in comparison with the traditional surgical method where wound infection, skin necrosis, and deep infection are observed in 11–34.1% of the cases [18]. The main reasons for these complications with traditional open surgery are the larger surgical incision and trauma, and wrinkling of the skin after suture. To ensure the torn sites are pulled together, the suture in traditional incision repair is usually knotted under plantar flexion, and this often requires plaster or brace fixation after surgery, which significantly exacerbates the skin folds on the incision site, affecting incision healing [31]. By using a limited incision, trauma is reduced, and suture tension of the soft tissue is reduced, as well as the degree of skin wrinkling. At the same time, the knots of anchored suture repair are wrapped in the tendon tissue by using absorbable line, followed by suturing the Achilles tendon sheath membrane, subcutaneous tissue and skin, which can effectively reduce postoperative adhesions and require fewer knot repairs than traditional incision. Therefore, we think that this method reduces the risk of postoperative incisional infection, skin necrosis, deep infection, and scar adhesion and reduces the occurrence of soft tissue complications. At present, there are no cases of soft tissue complications in the 23 patients who underwent this method.

Nevertheless, while minimally invasive repair has some advantages, the risk of Achilles tendon re-rupture is a shortcoming of this repair method. A biomechanical study reported that the mechanical resistance was decreased by 50% when using percutaneous repair compared with open repair [32], with a high risk of Achilles tendon re-rupture [20, 21]. The main reasons are because the torn sites are generally not revealed, and the biomechanical force of the broken end repair is weaker than the traditional incision repair, so the resistance strength will be weakened [33]. In terms of re-rupture, traditional open repair was thought to be the gold standard for acute Achilles tendon rupture treatment, with low postoperative re-rupture rates of 1.7–5% [34–36]. Nevertheless, the minimally invasive method presented here ensures that the repair strength of the torn sites is reliable, with a low risk of re-rupture since no patient showed re-rupture within a median follow-up of 22 months.

To enhance the biomechanical strength of the broken end repair, we designed the use of an anchor. Through anchoring, the tensile force carried by the distal end is transferred into the calcaneus to provide a stronger anchor. A biomechanical study from Sadoghi et al. [37] showed that traditional end-to-end repair can carry a maximum tension of 81–453 N, with an average of 222.7 N, while Beitzel et al. [38] showed that the maximum loading capacity of the suture anchors for Achilles tendon repair was 433 ± 84 N.

Minimally invasive percutaneous methods can have a relatively high sural nerve injury rate, which is reported to be 0–10% [39]. The sural nerve is not revealed during this type of surgery, and the stitches are mostly performed blind to the nerve, which leads to a direct risk of nerve damage. In addition, nerve friction caused by suture will also aggravate nerve damage. The method we used has larger trauma than percutaneous repair, but the choice of partial medial incision and operation of suture repair ends within the tendon tissue is completely different from the transverse penetration performed for most percutaneous repair techniques [27].

Moreover, most operations are under direct vision, so the incidence of sural nerve injury is low. Of the 23 patients in the present series, there were no cases of sural nerve injury, which is supported by previous studies [29, 30, 40].

Our method also effectively avoids extension of the posterior Achilles tendon. Traditional repair methods often need the greatest degree of plantar flexion of the ankle joint to reduce the tension of the suture. In addition, the Achilles tendon itself tends to creep. With increased postoperative stress, the degree of creep is exponentially declining [41], and extension of the Achilles tendon and contraction of weakness often happens after surgery. Therefore, some authors advocate that a certain amount of compressive stress should be ensured at the load of the torn sites when they are repaired [42]. We used an anchor tail suture and circuit suture to repair the torn sites to connect the proximal end of Achilles tendon to the muscle and maintain a good ductility, and as far as possible to ensure the Achilles tendon near the distal end, effectively avoiding Achilles tendon extension.

The method used by our hospital effectively reduces the chance of sutures cutting into the torn sites and ensures effective contact with the Achilles tendon fibers at the maximum extent, thus promoting healing of the torn sites. Acute Achilles tendon ruptures often have a caudal-like tear with irregular shape. Traditional end-to-end repair relies on sutures to pull the ends together, which will risk the suture cutting into the torn sites. The use of an anchor tail suture longitudinally through the tendon tissue and the circuit technique in the Achilles tendon repair at different layers can effectively reduce the suture cutting into the torn sites. At the same time, by anchoring, the tension load between the calcaneus and the muscle will produce a compressive stress, to ensure maximal effective contact with the Achilles tendon fibers, thus effectively promoting healing of the torn sites.

Compared with most minimally invasive repair techniques, only one anchoring nail was used in the present study and no special minimally invasive device was needed, which should reduce medical cost. Indeed, most techniques require the use of a special device such as the Ligadon, Achillon, or Dresden device [23, 29, 43, 44]. In addition, the use of the anchoring nail should provide a strong repair that should decrease the risk of treatment failure, again resulting in lower costs. Nevertheless, no direct comparison with other methods was performed in the present study and trials should be designed to compare the different techniques available. Unfortunately, because of the relative rarity of Achilles acute tear, such trials would be long and costly.

The present study is not without limitations. First, this is a retrospective non-controlled study with a relatively small number of patients and short follow-up. During follow-up, the strength of ankle plantar flexion and dorsiflexion needs to be recorded in a stretching test, and this should be compared with the traditional incision repair and percutaneous repair to obtain more objective and accurate long-term functional efficacy results. Secondly, the repair of the acute Achilles tendon rupture should be further supplemented with biomechanical experiments. Two anchors are usually used in repairing acute Achilles tendon rupture, and more are used for distal Achilles tendon rupture [41], but we used only one anchor. Whether one anchor can achieve good strength of Achilles tendon repair needs to be further biomechanically compared with the traditional incision repair and two-anchor repair. Finally, for elderly patients with severe osteoporosis, suture anchors may not provide a strong anchoring force.

## Conclusions

In conclusion, the main difference with previous methods is that this surgical method was improved to reduce the surgical incision complications, while relying on the force of the anchor to enhance the biomechanical stability of the torn ends. On the one hand, our method allows early rehabilitation exercises and short recovery because of the strengthened repair of the torn ends. On the other hand, reduction in the number of sutures results in smaller scars in the broken end and better flexibility of the Achilles tendon. When using the traditional open methods, a considerable portion of patients suffer from postoperative complications of soft tissues [18]. This is because the soft tissues are completely opened during surgery in order to reveal the Achilles tendon. Nevertheless, after the Achilles tendon is repaired, the incision often requires a long healing time due to heavy folds of the skin in incision closure. Hence, in this study, the soft tissues were only partially opened, which could significantly reduce the extent of folds and was beneficial to incision healing.

## Abbreviations

AOFAS: American Orthopedic Ankle Association
ROM: Range of motion
